# Strategic Ingestion of High-Protein Dairy Milk during a Resistance Training Program Increases Lean Mass, Strength, and Power in Trained Young Males

**DOI:** 10.3390/nu13030948

**Published:** 2021-03-15

**Authors:** Maryam Pourabbas, Reza Bagheri, Babak Hooshmand Moghadam, Darryn S. Willoughby, Darren G. Candow, Bradley T. Elliott, Scott C. Forbes, Damoon Ashtary-Larky, Mozhgan Eskandari, Alexei Wong, Frédéric Dutheil

**Affiliations:** 1 Department of Exercise Physiology, University of Tehran, Tehran 1961733114, Iran; Pourabbas.maryam@yahoo.com; 2 Department of Exercise Physiology, University of Isfahan, Isfahan 81746-73441, Iran; 3 Department of Exercise Physiology, Ferdowsi University of Mashhad, Mashhad 9177948974, Iran; b.hooshmand.m@gmail.com; 4 School of Exercise and Sport Science, University of Mary Hardin-Baylor, Belton, TX 76513, USA; dwilloughby@umhb.edu; 5 Faculty of Kinesiology and Health Studies, University of Regina, Regina, SK S4SOA2, Canada; darren.candow@uregina.ca; 6 Translational Physiology Research Group, School of Life Sciences, University of Westminster, London WC2N 5DU, UK; B.Elliott@westminster.ac.uk; 7 Department of Physical Education, Faculty of Education, Brandon University, Brandon, MB R7A6A9, Canada; forbess@brandonu.ca; 8 Nutrition and Metabolic Diseases Research Center, Ahvaz Jundishapur University of Medical Sciences, Ahvaz 61357-15794, Iran; damoon_ashtary@yahoo.com; 9 Department of Exercise Physiology, University of Birjand, Birjand 9717434765, Iran; Mozhgan.eskandari@birjand.ac.ir; 10 Department of Health and Human Performance, Marymount University, Arlington, VA 22207, USA; 11 Physiological and Psychosocial Stress, CNRS, LaPSCo, Université Clermont Auvergne, F-63000 Clermont-Ferrand, France; fred_dutheil@yahoo.fr; 12 Preventive and Occupational Medicine, Witty Fit, University Hospital of Clermont-Ferrand, CHU Clermont, F-63000 Clermont-Ferrand, France

**Keywords:** hypertrophy, body composition, milk, endocrine, resistance training

## Abstract

Background: We evaluated the effects of high-protein dairy milk ingestion on changes in body composition, strength, power, and skeletal muscle regulatory markers following 6 weeks of resistance training in trained young males. Methods: Thirty resistance-trained young males (age: 27 ± 3 years; training experience: 15 ± 2 months) were randomly assigned to one of two groups: high-protein dairy milk (both whey and casein) + resistance training (MR; *n* = 15) or isoenergetic carbohydrate (maltodextrin 9%) + resistance training (PR; *n* = 15). Milk and placebo were ingested immediately post-exercise (250 mL; 30 g protein) and 30 min before sleep (250 mL; 30 g protein). Before and after 6 weeks of linear periodized resistance training (4 times/week), body composition (bioelectrical impedance), strength, power, and serum levels of skeletal muscle regulatory markers (insulin-like growth factor 1 (IGF-1), growth hormone, testosterone, cortisol, follistatin, myostatin, and follistatin–myostatin ratio) were assessed. Results: The MR group experienced a significantly higher (*p* < 0.05) increase in lean mass, strength, and power (upper- and lower-body) than the PR group. Further, IGF-1, growth hormone, testosterone, follistatin, and follistatin–myostatin ratio were significantly increased, while cortisol and myostatin significantly decreased in the MR group than the PR group (*p* < 0.05). Conclusions: The strategic ingestion of high-protein dairy milk (post-exercise and pre-sleep) during 6 weeks of resistance training augmented lean mass, strength, power, and altered serum concentrations of skeletal muscle regulatory markers in trained young males compared to placebo.

## 1. Introduction

It is well known that the rates of muscle protein synthesis (MPS) are augmented after resistance training sessions when dietary proteins are ingested post-exercise in young adults [1,2]. With repeated training sessions, this could result in muscle hypertrophy and improvements in muscle performance (i.e., strength, power). Further, emerging evidence suggests that pre-sleep protein intake also increases muscular accretion and strength in young adults [3]. Dairy milk has a high digestible indispensable amino acid score (DIAAS) and contains a complement of whey (~20%) and casein proteins (~80%) that have both been shown to stimulate the mammalian target of rapamycin (mTOR) signaling pathway engaged in protein translation [4,5]. Whey proteins are rapidly absorbed and result in a transient surge in essential amino acid availability for MPS [6,7], while casein proteins are absorbed at a slower rate resulting in prolonged hyperaminoacidemia, possibly expanding the duration and capacity of MPS [6]. Theoretically, dairy milk, which contains both whey and casein proteins, should rapidly stimulate and prolong MPS following resistance training sessions leading to significant muscular alterations over time [8,9,10].

Research about the efficacy of diary milk ingestion during a resistance training program in young adults is limited. In young untrained males, the ingestion of dairy milk (35 g of protein) throughout 12 weeks of resistance training led to greater improvements in lean tissue mass and type I and II muscle fiber area, with trends for larger strength gains (for both extension and flexion of the knee) compared to those ingesting soy protein [11]. Furthermore, young untrained males who ingested chocolate milk (17 g of protein) following resistance training sessions for a period of 10 weeks experienced greater gains in lean tissue mass (although non-significant) from chocolate milk (1.6 ± 0.4 kg) compared to placebo (0.8 ± 0.5 kg) [12]. Collectively, these preliminary studies suggest that dairy milk can augment the physiological adaptations that result from resistance training in young untrained males. However, the effects in resistance-trained young males are unknown [13]. Moreover, because a higher protein intake may have a beneficial impact on body composition parameters in resistance-trained cohorts [14], the consumption of dairy milk with a high protein content may lead to positive body composition effects in this population.

The primary purpose of this research was to assess the effects of high-protein dairy milk ingestion vs. high-carbohydrate maltodextrin ingestion and resistance training on body composition and muscle performance (strength and power) in trained young males. It was hypothesized that high-protein dairy milk ingestion (post-exercise and pre-sleep) would lead to greater gains in lean mass and muscle performance following 6 weeks of resistance training compared to high-carbohydrate maltodextrin in trained young males. As a secondary purpose, we evaluated the effects of the intervention on serum concentrations of skeletal muscle regulatory markers, as muscle protein balance can be influenced by various hormones and myokines [15]. Indeed, the assessment of different anabolic and catabolic muscular regulatory markers might help in identifying mechanisms for potential favorable changes in body composition and muscular performance.

## 2. Materials and Methods

### 2.1. Participants

Thirty trained young males (age: 27 ± 3 years, body mass: 77.8 ± 5.5 kg, resistance training experience: 15 ± 2 months) were enrolled into the randomized controlled trial (RCT; Clinicaltrials.gov: NCT04509297). In addition, The Institute of Physical Education and Sports Sciences (Tehran, Iran) Human Subject Committee approved the study protocol (IR.SSRI.REC.1397.352). Participants were informed of the procedures, and their written informed consent was obtained before starting the study, in accordance with the Declaration of Helsinki. Inclusion criteria included being 18–35 years of age and were currently engaged in resistance training (≥1 year before the initiation of the study). Exclusion criteria included smoking, regular alcohol consumption, having pre-existing medical issues, being lactose sensitive or intolerant, and/or having ingested dietary supplements containing protein, creatine, or omega-3 fatty acids for 24 weeks before the start of the study. Furthermore, eligible participants were excluded if they were unwilling to fully adhere to the trial procedures. Physical activity and medical/health questionnaires were provided to each participant before the start of the study to confirm their health status. We inquired not to alter their regular lifestyle and habitual dietary intake patterns throughout the investigation. Baseline characteristics are shown in Table 1.

### 2.2. Study Design

**Figure 1:**
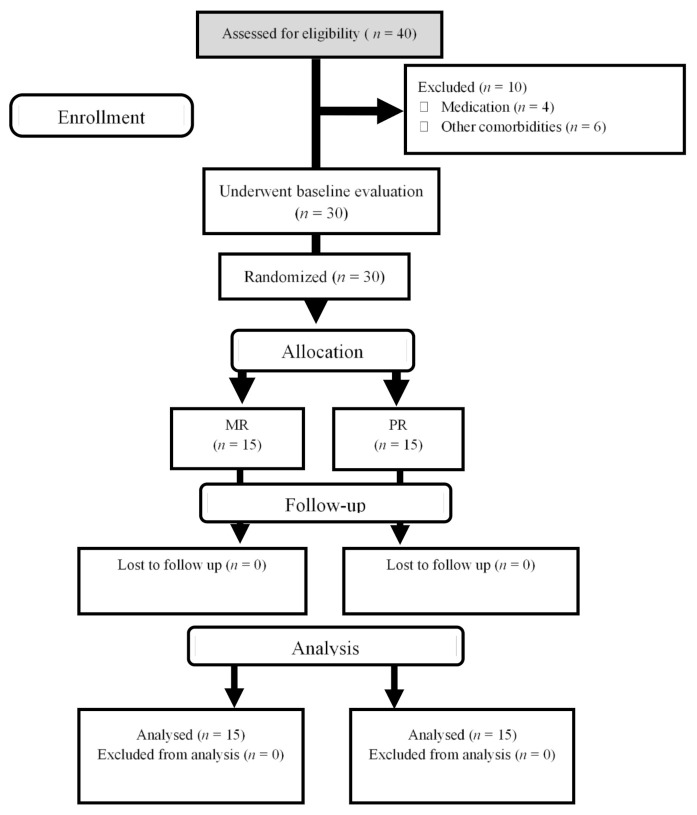
Flow diagram of study design.

The study involved a double-blind, randomized, placebo (control), and repeated-measures design (allocation ratio: 1:1). Before pre-training assessments, participants were acquainted with the experimental protocols. Subsequently, participants were randomly assigned to one of two groups: high-protein dairy milk + resistance training (MR; *n* = 15) or isoenergetic carbohydrate (placebo) + resistance training (PR; *n* = 15). The randomization assignment was stratified by digital technology (available at www.randomizer.org, accessed on 15 January 2018). A nutritionist at the university clinic, who was not a member of the core research team, assigned participants to their group and gave instructions on their nutritional interventions. Consequently, this individual could not be blinded. However, the mentioned nutritionist did not take part in data collection and/or analysis. Both participants as well as other members of research team (except the stated nutritionist) were blinded to the treatment allocation until the database was unlocked, and analysis of data was finalized. Measurements were made prior to and following 6 weeks of the nutritional intervention (high-protein dairy milk or placebo) and resistance training. Post-testing measurements occurred ≥72 h after the last resistance training session. All measurements (pre- and post-testing) were performed at approximately the same time each day (within ~1 h) and under the same environmental conditions (~20 °C and ~55% humidity). A flow diagram is shown in Figure 1.

### 2.3. Resistance Training Program

Participants participated in a supervised resistance training (split-routine) program 4 days per week for 6 weeks. Prior to training sessions, a 10-min warm-up (aerobic exercise and stretching) was completed. Following the warm-up, participants performed upper-body exercises 2 days per week (bench press, lateral pull-down, shoulder press, seated row, shoulder shrugs, chest flies, bicep curl, triceps press down, and abdominal curl) and lower-body exercises 2 days per week (leg press, back squat, step-ups, leg curls, back extension, calf raises, and abdominal curls). Upper- and lower-body exercises were performed on alternating days for the duration of the study. Participants performed 3 sets of 8–10 repetitions using 60–75% of baseline one-repetition maximum (1-RM) for weeks 1–3 and 3 sets of 4–8 repetitions using approximately 75–90% of baseline 1-RM for weeks 4–6. Rest intervals between exercises and sets was 2 min (Table 2) [16]. This periodized resistance training program was designed based off recommendations by the National Strength and Conditioning Association [17].

### 2.4. Nutritional Intervention

Participants ingested 250 mL of high-protein dairy milk—156 kcal, 30 g of mixture of whey (6 g) and casein (24 g), 0 g fat, 10 g carbohydrate; Kalleh Industry (Amol, Mazandaran, Iran), health license number: 49/14,996; formulation verified by a certificate of analysis from ViroMed Laboratory (Tehran, Iran)—or an iso-energetic placebo (maltodextrin 9%) beverage immediately following resistance training sessions and approximately 30 min prior to sleep. High-protein dairy milk was a commercial protein food product. This ingestion strategy was chosen because immediate post-exercise and pre-sleep protein ingestion have favorable effects on muscle mass and performance [3,11]. High-protein dairy milk and placebo were in opaque (masked) beverage containers and were similar in taste and smell (flavored with vanilla), texture, and appearance (beige color). All researchers and resistance training supervisors were blind to group allocations during the duration of the RCT. High-protein dairy milk and placebo ingestion started on the first day of the resistance training program and occurred in the presence of a resistance training supervisor. Pre-sleep ingestion was not directly monitored, but participants were required to return their empty containers prior to their next subsequent training session.

### 2.5. Body Composition

Prior to testing days, participants fasted for 12 h (water was permitted ad libitum). Immediately prior to measurements, participants voided their bladder. Body mass was determined with a digital scale (Seca, Hamburg, Germany) to the nearest 0.1 kg. Stature was quantified with a stadiometer (Race Industrialization, Shanghai, China) to the nearest 0.1 cm. Body mass index (BMI), fat mass, and lean mass were assessed by a multi-frequency bioelectrical impedance device (Inbody 770, Seoul, South Korea). The test–retest reliability of the bioelectrical impedance method was *r* = 0.96 to 0.99.

### 2.6. Blood

Standard procedures were utilized to collect fasting blood samples (10 mL) from the antecubital vein. Samples were then placed at room temperature for 15 min to form clots. The samples were then centrifuged at 3000 rpm for 10 min, and serum was stored at −80 °C for future analysis of ELISA kits for testosterone (abcam, ab108666, Cambridge, MA, USA; sensitivity: 0.07 ng/mL), Insulin-like growth factor 1 [(IGF-1); (abcam, ab100545, Cambridge, MA, USA; sensitivity: <0.2 ng/mL)], growth hormone [(GH); abcam, ab190811, Cambridge, MA, USA; sensitivity: 1.6 pg/mL), cortisol (CUSABIO, E05111h, Houston, TX, USA; sensitivity: 0.049 ng/mL), follistatin (CUSABIO, E08506h, Houston, TX, USA; sensitivity: 0.025 ng/mL), and myostatin (CUSABIO, E11300h, Houston, TX, USA; sensitivity: 0.312 ng/mL). All serum sample concentrations were measured in duplicate with a microplate reader (GDV, Rome, Italy) at a wavelength of 450 nm. The intra and inter-assay CV for IGF-1 was <10 and <12%, testosterone 5.8 and 10.5%, GH 3.6% for both, cortisol < 8%, < 10% follistatin, and myostatin < 15%.

### 2.7. Strength 

Estimated 1-RM strength for the back squat and bench press occurred 24 h following body composition assessment and blood sampling for both pre- and post-training testing. An additional 1-RM strength test took place during week 3 (mid-point) to further control training load. Prior to the start of the 1-RM test, participants were instructed to refrain from alcohol for 48 h, caffeinated drinks for 12 h, and food and drink for 2 h. It should be mentioned that water consumption was permitted ad libitum. To determine their estimated 1-RM, participants performed two sets of <10 repetitions to volitional fatigue with increasing loads [18]. Their heaviest load and highest number of repetitions performed were then used to determine their 1-RM using the following Equation (1) [19]. The intraclass correlation coefficient was *r* = 0.95 to 0.97.

1-RM = load ÷ (1.0278 − 0.0278 × repetitions performed)(1)

### 2.8. Power

Twenty-four hours after the determination of estimated 1-RM strength, anaerobic power for the upper and lower body was assessed via Monark Wingate cycle ergometry (Monark model 894e, Vansbro, Sweden) as previously described [20,21]. Briefly, participants cycled or cranked against a pre-determined resistance (7.5% of body mass for the lower body test and 5.5% of body mass for the upper body test) as fast as possible for 10 s [20]. Peak power output was documented in real time during the test, utilizing a Monark Anaerobic test software (v. 3.0, Monark Exercise AB, Vansbro, Sweden) [20].

### 2.9. Diet

Participants filled out 3-day food records prior to and immediately following the intervention to determine changes in total energy (kcal) and macronutrient intake over time. Participants were instructed to record all food items (independent of milk or placebo ingestion) for 2 weekdays and 1 weekend day. Diet Analysis Plus version 10 (Cengage, Boston, MA, USA) was used to analyze food records [18]. 

### 2.10. Statistical Analysis

Based on data from previous studies evaluating muscular outcomes following resistance training combined with different protein supplementation in young adults [20,22], it was calculated that 12 participants per group would provide 80% power (two-sided α = 0.05) to detect 7% between group changes in lean mass and muscular strength. The normality of data was confirmed using the Shapiro–Wilk test. The student’s *t*-test was used for group comparisons at baseline. A two × two ANOVA with repeated measures (time [baseline vs. 6 weeks] × group [MR vs. PR]) was used to determine the differences between the treatments over time. Pearson’s linear regressions were performed with 95% confidence interval (CI). Reported F values refers to group × time interactions. Statistical significance was set at *p* < 0.05. Cohen’s *d* effect size (ES) was calculated as post-training mean minus pre-training mean / pooled pre-training standard deviation means [23]. All analyses were performed using SPSS (v. 24.0, IBM; Chicago, IL, USA). 

## 3. Results

Between January and May of 2018, we screened 40 trained young males. Of these, 30 qualified for baseline testing and were consequently randomized to either the MR or the PR groups. No participants dropped out after randomization. Data are shown for the 30 participants that finished our 6-week study; 15 participants were in both the MR and PR groups.

### 3.1. Compliance, Adverse Effects, and Diet

Compliance with the nutritional intervention (milk and placebo) and the resistance training program was 100%. There were no adverse events reported from either nutritional intervention or the resistance training program. There were no significant differences at baseline between groups (Table 3). There were significant group × time interactions for total energy, and absolute and relative protein intake (*p* < 0.001) (Table 3). Participants in the MR group increased total energy (MR: Δ 285.4 kcal/day, 95% CI: 204.7 to 366.1, *d* = 1.3, F = 29.7), absolute protein (MR: Δ 63.7 g/day, 95% CI: 54.8 to 72.6, *d* = 6.1, F = 102.7), and relative protein intake (MR: Δ 0.8 g/kg/day, 95% CI: 0.6 to 0.9, *d* = 4.3, F = 87.1) over time, with no changes in the PR group. The larger elevation in total energy in the MR group was entirely due to the dairy milk intervention (Table 3). 

### 3.2. Body Composition

**Figure 2:**
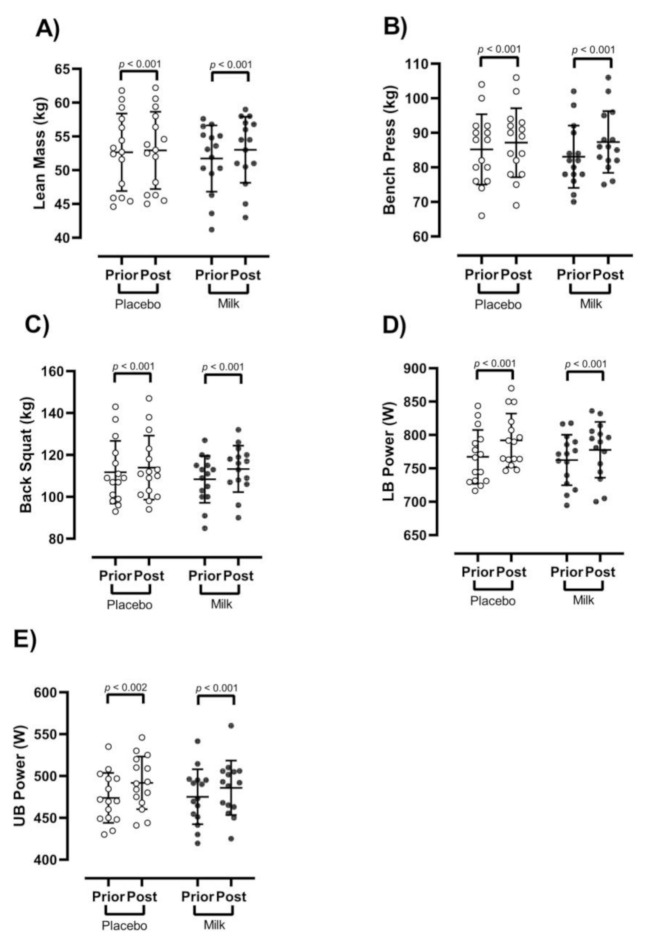
Effect of resistance training and groups on training-induced changes. (**A**) Lean mass (kg), (**B**) bench press (kg), (**C**) back squat (kg), (**D**) lower body power—LB power, (W), (**E**) upper body power—UB power, (W). *n* = 15 per group, error bars represent standard deviation, open white circles indicate placebo group, closed black circles indicate high-protein diary milk ingestion group.

There were significant group × time interactions for body mass (*p* < 0.001, F = 34.5) BMI (*p* < 0.001, F = 20.3), and lean mass (*p* < 0.001, F = 98.8), (Figure 2A). Compared to the PR, the MR group experienced superior changes in body mass (MR: Δ 2 kg, 95% CI: 1.6 to 2.3, *d* = 0.3; PR: Δ 0.8 kg, 95% CI: 0.6 to 1, *d* = 0.1), BMI (MR: Δ 0.4 kg.m^−2^, 95% CI: 0.3 to 0.5, *d* = 0.2; PR: Δ 0.1 kg.m^−2^, 95% CI: 0.1 to 0.2, *d* = 0.06), and lean mass (MR: 1.3 kg, 95% CI: 1 to 1.5, *d* = 0.2; PR: Δ 0.2 kg, 95% CI: 0.1 to 0.3, *d* = 0.04). There were no changes in fat mass between groups over time (*p* > 0.05), (Table 4).

### 3.3. Strength and Power

There were significant group × time interactions for bench press (*p* < 0.001, F = 57.5), (Figure 2B) and back squat strength (*p* < 0.001, F = 73.5), (Figure 2C), as well as lower-body power (*p* = 0.001, F = 12.6), (Figure 2D) and upper-body power (*p* = 0.030, F = 5.2), (Figure 2E). Compared to the PR, the MR group had larger changes in bench press strength (MR: Δ 4.2 kg, 95% CI: 3.7 to 4.7, *d* = 0.4; PR: Δ 1.9 kg, 95% CI: 1.4 to 2.3, *d* = 0.2), back squat strength (MR: Δ 4.9 kg, 95% CI: 4.4 to 5.4, *d* = 0.4; PR: Δ 2.2 kg, 95% CI: 1.7 to 2.6, *d* = 0.1), upper-body power (MR: Δ 17.9 Watts, 95% CI: 14.5 to 21.3, *d* = 0.5; PR: Δ 10.6 Watts, 95% CI: 4.8 to 16.5, *d* = 0.3), and lower-body power (MR: Δ 24.7 Watts, 95% CI: 20.4 to 29.1, *d* = 0.6; PR: Δ 15.3 Watts, 95% CI: 11.6 to 18.9, *d* = 0.3).

### 3.4. Hormones and Myokines

**Figure 3:**
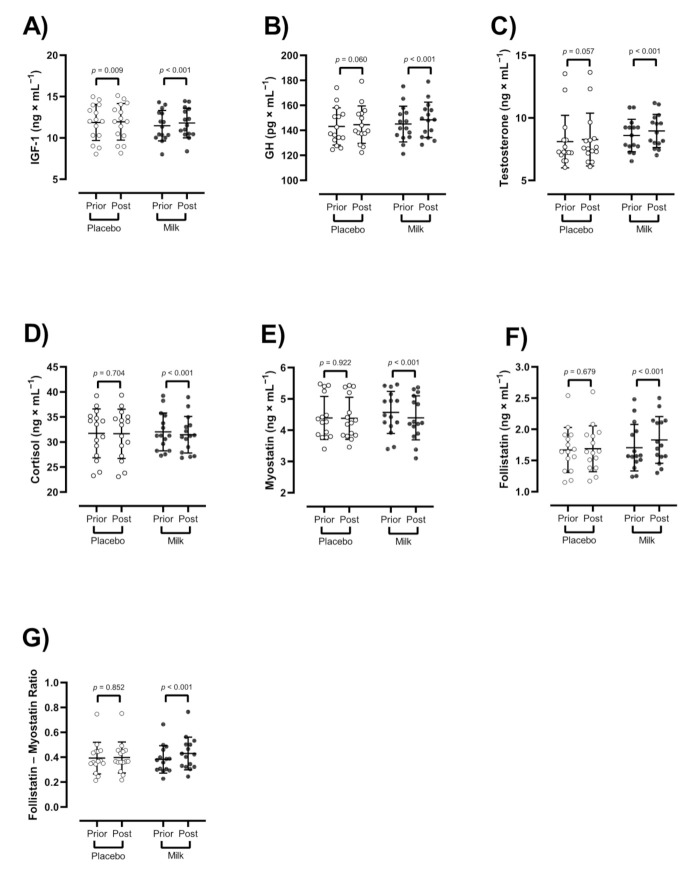
Effect of resistance training and groups on endocrine markers. (**A**) IGF-1 (ng × mL^−1^), (**B**) GH (pg × mL^−1^), (**C**) testosterone (ng × mL^−1^), (**D**) cortisol (ng × mL^−1^), (**E**) myostatin (ng × mL^−1^), (**F**) follistatin (ng × mL^−1^), (**G**) follistatin–myostatin ratio. *n* = 15 per group, error bars represent standard deviation, open white circles indicate placebo group, closed black circles indicate high-protein diary milk ingestion group.

There were significant group × time interactions for IGF-1 (*p* < 0.001, F = 17.4), (Figure 3A), GH (*p* = 0.043, F = 4.4), (Figure 3B), testosterone (*p* = 0.023, F = 5.7), (Figure 3C), cortisol (*p* = 0.004, F = 9.9), (Figure 3D), myostatin (*p* = 0.005, F = 9.4), (Figure 3E), follistatin (*p* = 0.009), F = 7.7), (Figure 3F), and follistatin–myostatin ratio (*p* = 0.003, F = 10.7), (Figure 3G). IGF-1 was significantly enhanced in both groups over time (MR: Δ 0.3 ng/mL, 95% CI: 0.2 to 0.4, *d* = 0.1; PR: Δ 0.07 ng/mL, 95% CI: 0.02 to 0.1, *d* = 0.03), with significantly larger changes in the MR group (*p* = 0.001). In addition, GH (MR: Δ 3.3 pg/mL, 95% CI: 2 to 4.6, *d* = 0.2), testosterone (MR: Δ 0.3 ng/mL, 95% CI: 0.2 to 0.4, *d* = 0.2), and follistatin–myostatin ratio (MR: Δ 0.04 ng/mL, 95% CI: 0.02 to 0.07, *d* = 0.4) significantly increased and cortisol significantly decreased in the MR group over time (MR: Δ −0.6 ng/mL, 95% CI: −0.9 to −0.2, *d* = 0.1), with no changes in the PR group (*p* > 0.05). 

### 3.5. Correlations

**Figure 4:**
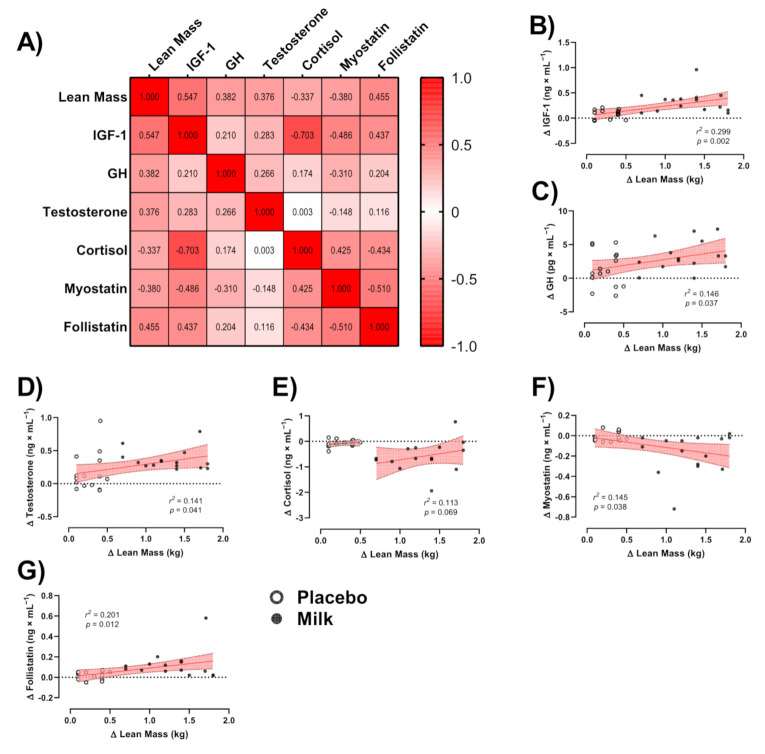
Relationships between change in lean mass (Δ lean mass (kg)) and change in endocrine markers (Δ (hormone)). (**A**) Correlation matrix of Δ lean mass and endocrine markers, *r* values as shown. Key indicates magnitude of r (red = −1 or 1, white = 0). (**B**–**G**) linear regression (Pearson’s) of Δ (hormone) as a function of Δ lean mass (kg). Linear regression indicated by solid red line, 95% confidence intervals indicated by shaded red zones. Open white circles indicate placebo group; closed black circles indicate high-protein diary milk ingestion group.

To assess any potential relationships between training-induced changes in lean mass (Δ lean mass) and changes in hormones examined (Δ hormone, independently of MR or PR group), initially, a correlation matrix was generated (Figure 4A). Cortisol and myostatin concentrations showed weak negative relationships with Δ lean mass, whilst GH and testosterone concentrations showed a weak positive relationship, and IGF-1 and follistatin concentrations showed moderate positive relationships. For linear regression of individual Δ (hormone) as a function of Δ lean mass, data were examined by the extra sum-of-squares F test to first consider if pooled data could be considered as a single model. All data except for Δ cortisol concentrations (*p* = 0.030) were considered as a single group (Figure 4B–G). Δ IGF-1, GH, testosterone, myostatin, and follistatin concentrations showed significant direct linear relationships with training-induced changes in lean mass (Δ IGF-1, *p* = 0.002; Δ GH, *p* = 0.037; Δ testosterone, *p* = 0.041; Δ myostatin, *p* = 0.038; follistatin, *p* = 0.012).

## 4. Discussion

This was the first study to investigate the effects of high-protein dairy milk ingestion (post-exercise: 30 g protein and pre-sleep: 30 g protein) during a resistance training program in trained young males already ingesting ~1.5 g/kg/day of protein. High-protein dairy milk ingestion increased total energy and dietary protein intake (~2.3 g/kg/day), which subsequently augmented muscle accretion, muscle performance (strength and power), and altered follistatin, IGF-1, GH, testosterone, myostatin, cortisol, and follistatin–myostatin ratio concentrations compared to an isoenergetic non-protein placebo. There were no adverse events reported from either nutritional intervention or the resistance training program. From a programming and scheduling perspective, high-protein dairy milk ingestion immediately after resistance training sessions and shortly before sleep are two effective strategies that resistance-trained young males can adopt to increase total energy intake and meet the highest recommended total daily protein quantity to ingest during a resistance training program (~2.2 g/kg/day) to maximize muscle accretion [24].

The greater enhancement in lean mass and strength from post-exercise high-protein dairy milk ingestion compared to placebo indirectly supports previous findings in young males. Hartman et al. [11] showed that post-exercise dairy milk ingestion for 12 weeks significantly increased fat- and bone-free mass (lean mass) and type I and II muscle fiber cross-sectional area, with a trend for superior gains in lower-body strength (leg press and knee extension–flexion) compared to those ingesting soy protein (placebo). Three-day food records showed that individuals consuming dairy milk had higher energy (kcal) intake after the 12 weeks of training (pre: 12.6 ± 0.9 MJ/day, post: 13.5 ± 1.2 MJ/day) compared to a reduction for those consuming placebo (pre: 13.0 ± 0.8 MJ/day, post: 12.7 ± 0.7 MJ/day), but this did not achieve statistical significance. Previous research has shown that post-exercise milk ingestion increases the rates of MPS in young adults [22]. With repeated training sessions, it may be possible that the summation of acute increases in MPS and additional energy intake contributed to the significant increases in muscular accretion as well as strength over time across studies. However, rates of MPS were not assessed in the present study so speculation remains. 

A unique aspect of the present study was that participants ingested the other 50% of their milk requirement 30 min before sleep. Previous research has demonstrated that pre-sleep protein consumption has favorable effects on MPS, accretion, and strength in young males. For example, young healthy males who performed an acute bout of resistance training and then ingested protein (casein; 40 g) 30 min before sleeping experienced greater increases in whole-body protein synthesis and net muscle protein balance compared to those who ingested placebo [25]. Furthermore, Snijders et al. [26] showed that ~28 g of protein (casein) immediately prior to sleep during a resistance training program (3×/week for 12 weeks) led to greater gains in knee extensor muscle cross-sectional area, type II fiber size, and total body muscle strength in young males compared to placebo. Three-day food records revealed no differences in the total intake of energy over time between groups (protein: pre 11.5 ± 0.7 MJ/day, post 11.4 ± 0.7 MJ/day; placebo: pre 12.0 ± 0.7 MJ/day, post 10.8 ± 0.6 MJ/day). Pre-sleep protein was adequately digested and absorbed possibly resulting in net muscle protein accretion [25,26]. While the absorption kinetics and mechanistic effects of pre-sleep protein (primarily casein) were not assessed in the present study, the strategic ingestion of high-protein dairy milk 30 min prior to sleep may have added to the enhancement in lean mass and strength over time. Unfortunately, no comparison between post-exercise and pre-sleep protein was made in this study, so conclusions cannot be made regarding the influence that each timing strategy provided.

Myostatin is a strong negative regulator of muscle mass. Myostatin binds to muscle activin type II receptors to activate the intracellular mediator SMAD 2/3 pathway [27]. Conversely, follistatin is an antagonist of the myostatin receptor, with both paracrine and autocrine effects, which lessens myostatin activity and enhances muscle mass [28]. In the presence of follistatin, myostatin is unable to bind to its receptor, and its atrophic actions are impeded. Previous research notes the converse relationship between myostatin and muscle mass gains in humans [18]. A growing body of literature has been published on the effects of resistance training on follistatin and myostatin in humans. For instance, our lab has shown a significant drop in serum myostatin and a significant rise in follistatin following 8 weeks of whole-body resistance training in middle-aged men [18]. More recently, 8 weeks of variable and constant whole-body free weight resistance training significantly increased follistatin, while decreasing myostatin in young females [29]. These findings are in line with previous findings of other studies that used moderate to heavy resistance training [30,31,32,33,34]. The present study utilized a high volume heavy resistance training program, which involved larger muscle groups, and subsequently, high-protein dairy milk ingestion led to a significant fall in myostatin compared to a non-significant decrease in the placebo group. These differences may be associated with myostatin’s responsiveness to glucocorticoids [35], since it appears to be downregulated in response to a drop in cortisol, which was observed in the high-protein dairy milk ingestion group. Therefore, it appears that post-exercise and pre-sleep protein can influence the myokine response to heavy resistance training. Further, high-intensity physical activity increases IGF-1 production [36,37]. In support of previous literature, including a recent systematic review and meta-analysis, there was an increase in IGF-1 with resistance training [38]. The stimulation of exercise-induced GH could lead to an enhancement of IGF-1 [39], supporting our findings. In addition, in the present research, there was a significant elevation in testosterone in the high-protein dairy milk ingestion group, which may be mediated by post-exercise and pre-sleep high-protein dairy milk ingestion [40]. Potential mechanisms for training-induced changes in lean mass for both groups might be related to changes in endocrine hormones, as we showed negative relationships between cortisol and myostatin with Δ lean mass; while there were positive relationships for this variable with GH, testosterone, IGF-1, and follistatin concentrations. However, correlation is not causation, thus these correlations need to be interpreted with caution. 

This investigation is limited by the absence of measurements of skeletal muscle anabolism (mTORC1 signaling, MPS), which would have assisted in the explanation of certain outcomes. However, it has been proposed that promotions in circulating levels of signaling molecules amplifies the likelihood of a receptor interaction and, therefore, a biological effect within skeletal muscle [41,42]. Additionally, the effects of post-exercise lower-protein dairy milk on lean mass and strength in young populations have been previously described [12,43]; therefore, we did not compare high-protein dairy milk to lower-protein dairy milk. Although we used 3-day food records, a valid and reliable method to determine total energy and macronutrient intake [44,45,46], it could be argued that controlling habitual (daily) dietary intake for 6 weeks would have strengthen our study design. However, a large portion of studies in the field of nutrition do not use this method, as controlling every single meal for a high number of subjects during several weeks/months is extremely challenging and impractical. Another limitation of our study is that we did not use dual-energy X-ray absorptiometry, the gold standard technique for body composition measurement; yet, prior studies have shown that multi-frequency bioelectrical impedance is a valid and reliable method [47,48]. Moreover, recent research indicated that multi-frequency bioelectrical impedance is a viable alternative to dual-energy X-ray absorptiometry for tracking changes in body composition in resistance-trained men [49], our study’s population.

In conclusion, the strategic ingestion of high-protein dairy milk (post-exercise and pre-sleep; 60 g protein in total) appears to be a viable and well-tolerated strategy to increase total energy intake and augment lean mass and muscle performance in young resistance-trained males who were already ingesting 1.5 g/kg/day of protein. Furthermore, high-protein dairy milk ingestion created a more favorable anabolic environment, as indicated by an elevation in the concentrations of anabolic hormones and myokines.

Results of the present study have application for the design of optimal dietary protein ingestion strategies in young, trained males. The ingestion of high-protein dairy milk immediately post-exercise and shortly before sleep can be easily adopted by young, trained males to help achieve dietary protein goals for muscle and performance improvements.

## Figures and Tables

**Table 1:** Descriptive characteristics of participants’ values represent mean, standard error in brackets.

Descriptive	Milk	Placebo	*p*-Value
Age (years)	26 (1)	27 (1)	0.495
Stature (cm)	178.6 (1.1)	177.1 (1.5)	0.429
Body mass (kg)	77.1 (1.3)	78.4 (1.5)	0.511
BMI (kg × m^−2^)	24.2 (0.5)	25.1 (0.8)	0.312
Lean Mass (kg)	51.7 (1.2)	52.6 (1.4)	0.638
Fat mas (kg)	16.4 (0.3)	16.8 (0.3)	0.328
Bench press (kg)	83 (2.3)	85.2 (2.6)	0.549
Squat (kg)	108.4 (2.8)	111.7 (3.8)	0.496
Lower body power (watt)	767.1 (10.4)	762.4 (9.7)	0.741
Upper body power (watt)	473.7 (7.6)	475.1 (8.4)	0.905
IGF-1 (ng × mL^−1^)	11.4 (0.4)	11.8 (0.5)	0.587
GH (pg × mL^−1^)	145 (3.6)	143.1 (3.8)	0.721
Testosterone (ng × mL^−1^)	8.5 (0.3)	8.1 (0.5)	0.450
Cortisol (ng × mL^−1^)	32 (0.9)	31.7 (1.2)	0.852
Myostatin (ng × mL^−1^)	4.5 (0.1)	4.3 (0.1)	0.484
Follistatin (ng × mL^−1^)	1.7 (0.1)	1.6 (0.1)	0.786
Follistatin–Myostatin ratio (ng × mL^−1^)	0.3 (0.02)	0.4 (0.03)	0.812

*p* values indicate unpaired sample *t*-test (placebo vs. milk), *n* = 15 per group. Abbreviations: BMI, body mass index; IGF-1, insulin-like growth factor 1; GH, growth hormone; ng × mL^−1^, nanogram per milliliter; pg × mL^−1^, picogram per milliliter. Data represent mean (standard error of mean).

**Table 2:** Resistance training procedures.

		Weeks 1–2–3	Weeks 4–5–6	Rest
Day 1 and 3	Bench press	12, 10, 8	8, 6, 4	1.5–2 min
	Lateral pull	12, 10, 8	8, 6, 4	1.5–2 min
	Shoulder press	12, 10, 8	8, 6, 4	1.5–2 min
	Seated rows	12, 10, 8	8, 6, 4	1.5–2 min
	Shoulder shrugs	3 × 12	3 × 8	30–60 sec
	Chest Flies	3 × 12	3 × 8	30–60 sec
	Bicep curl	3 × 12	3 × 8	30–60 sec
	Triceps Press down	3 × 12	3 × 8	30–60 sec
	Abdominal curls	125 weighted reps	125 weighted reps	none
Day 2 and 4	Leg press	12, 10, 8	8, 6, 4	1.5–2.5 min
	Back squats	12, 10, 8	8, 6, 4	1.5–2.5 min
	Step-ups	12, 10, 8	8, 6, 4	30–60 sec
	Leg curls	12, 10, 8	8, 6, 4	30–60 sec
	Back extension	3 × 12	3 × 8	30–60 sec
	Calf raises	3 × 12	3 × 8	30–60 sec
	Abdominal curls	200 unweighted reps	200 unweighted reps	none

**Table 3:** Energy and macronutrients (mean ± SD).

Variables	Group	Pre-Training	Post-Training	*p*-Value
Energy (kcal/day)	Milk	2623.8 ± 107.4	2909.2 ± 109.2 *^, #^	<0.001
	Placebo	2618.6 ± 104.9	2653.3 ± 109.1	
Absolute Protein (g/day)	Milk	114.4 ± 7.7	178.2 ± 13 *^, #^	<0.001
	Placebo	112.8 ± 11.7	114.2 ± 11.3	
Relative Protein (g/kg/day)	Milk	1.5 ± 0.1	2.3 ± 0.2	<0.001
	Placebo	1.4 ± 0.1	1.4 ± 0.2	
Absolute Fat (g/day)	Milk	95.2 ± 5.4	97.5 ± 4.4	0.285
	Placebo	94 ± 6.2	95.4 ± 4.6	
Relative Fat (g/kg/day)	Milk	1.2 ± 0.1	1.2 ± 0.08	0.323
	Placebo	1.2 ± 0.1	1.2 ± 0.08	
Absolute Carbohydrate (g/day)	Milk	327.1 ± 17	329.6 ± 15.4	0.385
	Placebo	330.2 ± 16	334.3 ± 14	
Relative Carbohydrate (g/kg/day)	Milk	4.2 ± 0.3	4.1 ± 0.3	0.919
	Placebo	4.2 ± 0.3	4.2 ± 0.3	

Abbreviations: kcal/day, kilocalorie/day; g/day; gram/day. * different from baseline; ^#^ different from placebo. *p*-value refers to between-group changes.

**Table 4:** Body composition characteristics (mean ± SD).

Variables	Group	Pre-Training	Post-Training	*p*-Value
Body mass (kg)	Milk	77.1 ± 5.1	79.1 ± 5.3	<0.001
	Placebo	78.5 ± 5.9	79.3 ± 5.9	
Body mass index (kg/m^2^)	Milk	24.2 ± 1.8	24.6 ± 1.8	<0.001
	Placebo	25.1 3.1	25.3 ± 3	
Fat mass (kg)	Milk	16.4 ± 1.1	16.3 ± 1.1	0.432
	Placebo	16.8 ± 1.3	16.8 ± 1.3	

*p*-value refers to group × time interaction.

## Data Availability

The data presented in this study are available on request from the corresponding author. The data are not publicly available due to restrictions.
